# Hidden in plain sight: the fourth UN High-Level Meeting on the Prevention and Control of Noncommunicable Diseases and the Promotion of Mental Health and Well-being missed the mark on chronic pain and mobility impairment

**DOI:** 10.7189/jogh.16.03009

**Published:** 2026-04-17

**Authors:** Andrew M Briggs, Neil Betteridge, Fiona M Blyth, Karsten E Dreinhöfer, Deborah Kopansky-Giles, Lyn M March, Andrew S C Rice, Anthony D Woolf

**Affiliations:** 1 Curtin School of Allied Health, Curtin University, Perth, Australia; 2 Global Alliance for Musculoskeletal Health, Institute of Bone and Joint Research, Kolling Institute, University of Sydney Faculty of Medicine and Health, University of Sydney, Sydney, Australia; 3 Neil Betteridge Associates, London, UK; 4 Sydney School of Public Health, Faculty of Medicine and Health, University of Sydney, Sydney, Australia; 5 International Association for the Study of Pain, Washington, D.C., USA; 6 Center for Musculoskeletal Surgery, Charité University Berlin, Berlin, Germany; 7 Department of Orthopaedics and Traumatology, Medical Park Berlin Humboldtmühle, Berlin, Germany; 8 Department of Research and Innovation, Canadian Memorial Chiropractic College, Toronto, Canada; 9 Department of Family & Community Medicine, University of Toronto, Toronto, Canada; 10 Kolling Institute and Florance and Cope Professorial Department of Rheumatology, Royal North Shore Hospital and University of Sydney Faculty of Medicine and Health, Sydney, Australia; 11 Department of Surgery and Cancer, Imperial College, Faculty of Medine, London, UK; 12 Department of Clinical Sciences (Orthopaedics), Lund University, Malmö, Sweden

## Abstract

The fourth United Nations High-Level Meeting on the Prevention and Control of Noncommunicable Diseases (NCDs) and Promotion of Mental Health and Well-Being developed a timely Political Declaration intended to accelerate progress among Member States towards the targets of Sustainable Development Goal 3.4. The Declaration emphasises appropriate cross-cutting actions for prevention and control of NCDs generally, yet over the consultation period, it evolved to include a range of specific diseases beyond the SDG 3.4 targets. The Declaration omits explicit attention to chronic pain and mobility impairment due to musculoskeletal conditions, despite their substantial contribution to disability and NCD multimorbidity across the life course. The omission creates a missed opportunity for health gain across NCDs, particularly in low- and middle-income countries experiencing rapid population ageing and expansion, and it disrupts implementation of aligned global technical products. Chronic pain affects a substantial proportion of the global population, and musculoskeletal conditions are among the leading causes of years lived with disability, yet global NCD policy frameworks continue to privilege mortality reduction over morbidity. Embedding prevention and management of chronic pain and mobility impairment within integrated NCD and mental health strategies offers a pathway to reduce avoidable disability and improve global healthy life years beyond 2030.

On 25 September 2025, United Nations Member States met to endorse a Political Declaration on the prevention and control of NCDs and promotion of mental health and well-being [1]. This fourth High-Level Meeting (UNHLM4) built on three earlier meetings, held in 2011, 2014, and 2018, to advance efforts on prevention and control of NCDs. The 2025 Declaration reaffirmed commitments for action on NCDs and promotion of mental health over the next five years, with the objective to renew and accelerate efforts by Member States towards achieving targets of the health-related Sustainable Development Goal (SDG) 3.4 by 2030.

Political Declarations and Resolutions are important levers to advancing global health priorities. While they may not be adopted into law by countries, they serve to build a consensus among Member States for action on a health issue. For example, the first UN Political Declaration on antimicrobial resistance (AMR) in 2016 catalysed unprecedented attention to this global health issue [2]. By March 2018, 100 countries had prepared an AMR plan, increasing to 178 by November 2023 [3]. The UNHLM4 Political Declaration is important and timely, given that the burden of NCDs continues to rise and the world remains off track to achieve the agreed targets for SDG 3.4, particularly among the low- and middle-income countries (LMICs) [4,5]. Furthermore, the current climate of uncertainty around development assistance for health underscores the need to double down on commitments to arrest the rising NCD burden and to contain avoidable health losses and costs. The Declaration considered a range of NCDs, yet did not mention chronic pain or mobility impairment, which are common sequelae across the NCDs.

Here, we reflect on our combined experiences in health policy and systems research, public health and clinical practice and the global advocacy initiatives prioritised by the Global Alliance for Musculoskeletal Health (G-MUSC) and the International Association for the Study of Pain (IASP) [6–13]. Drawing on evidence and experience while evaluating the initial and final versions of the Political Declaration, we argue that by not integrating prevention and control of chronic pain (that is, chronic primary pain, or chronic pain associated with other conditions; *i.e.* chronic secondary pain, as classified by ICD-11 [14]) and supporting mobility through action on musculoskeletal conditions (*e.g.* arthritis, osteoporosis, back pain), the recognitions and commitments affirmed in the Declaration fall short to advance global health. Specifically, their omission misses a major opportunity to contain health and economic losses attributed to these health conditions and the other NCDs across the life course, and to close the gap between life expectancy and healthy life years driven by these conditions [5].

While the Declaration appropriately identifies important cross-cutting strategies and actions, such as attention to the modifiable risk factors for NCDs (tobacco use, harmful use of alcohol, unhealthy diets, physical inactivity and air pollution), the failure to recognise chronic pain and mobility impairment related to musculoskeletal conditions as cross-cutting issues relevant to the prevention and control of NCDs and mental health conditions, especially in older adults within the current UN Decade of Healthy Ageing [15], is an oversight. The Declaration points to the increasing gap between life expectancy and healthy life years for older people [1], yet remains silent on the major contributors to years lived with disability (YLDs), the condition groups for which the greatest number of people (1.7 billion) could benefit from rehabilitation, and one of the most frequent NCD groups in multimorbidity presentations – chronic pain conditions and musculoskeletal conditions [5,16,17].

Chronic pain is a common experience across most NCDs [18]. It affects at least 30% of the population worldwide [19,20], and it frequently has a bidirectional relationship with mental health conditions. Chronic pain is experienced more frequently by older people, women and people living in rural areas, and its prevalence is rising [19–21]. In 2023, musculoskeletal conditions accounted for 16% of YLDs globally, second only to mental health disorders (19%), and contributed the largest share (22%) of YLDs among people aged 55–69 years [22]. Trajectories for growth in cases of all musculoskeletal conditions to 2050 are steepest in LMICs, where population expansion and ageing rates are accelerating [7]. However, these burden estimates should be considered in the context of uncertainties related to scarce or absent primary data from some countries, cross-cultural differences in the perception of pain, and the application of uniform disability weights across countries [23].

While the prevalence of chronic pain and chronic musculoskeletal conditions increases with age, children and young adults also experience a high burden [24], including injury sequelae, which for many become lifelong and mutually reinforcing experiences. Chronic pain and mobility impairment impact physical functioning and mental well-being, manifesting in reduced participation in play, education and work, premature retirement and reduced accumulated wealth, particularly in the most vulnerable groups (children, older people, and people experiencing intersectoral disadvantage). Chronic pain and musculoskeletal conditions also increase the risk of developing other NCDs such as diabetes, cardiovascular disease and dementia (*e.g.* a 17% increased risk, based on meta-analysis), and exacerbate the risk of mortality from NCDs by two or three times [25–28]. In response to this global burden, the World Health Organization (WHO) has developed several technical products to support systems strengthening for prevention and control of chronic pain and musculoskeletal conditions for children, adults and older adults [29–32].

The UNHLM4 Political Declaration expresses Member States’ commitment to implement a priority set of evidence-based, cost-effective, and affordable actions, represented around six themes (**Figure 1**). These themes are relevant to all NCDs and resonate with a critical whole-of-government and whole-of-society approach to arresting the NCD burden [33]. From this perspective, it made sense for the draft-zero of the Declaration to be framed as largely agnostic to disease classification, yet reasonable that the ‘four main NCDs’ (cancer, diabetes, heart disease and chronic lung disease) were mentioned, consistent with the indicators for SDG 3.4 and with extant monitoring and systems strengthening technical products for NCDs which have historically focused on avoidable mortality, rather than arresting morbidity [34,35]. However, over the five-month consultation period among Member States and civil society, many more discrete NCDs were added to the Declaration text (**Figure 1**), including suicide and self-harm. The reasons for this shift remain unclear, but likely reflect diverse priorities and political economies among Member States, negotiation dynamics including civil society advocacy efforts, and the linking of individual NCDs to the ‘four main NCDs’ aligned with the SDG target 3.4 to reduce premature mortality, rather than morbidity. Further, classification of chronic primary pain was only introduced in ICD-11 in 2022 [14], so diffusion to policy remains nascent.

**Figure 1:**
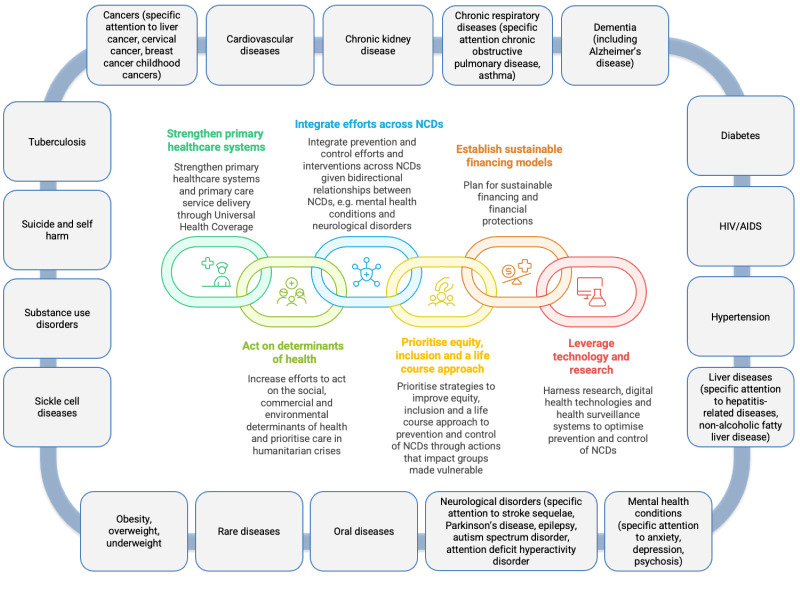
Visual summary of the six thematic areas in the UNHLM4 Political Declaration relevant to the prevention and control of NCDs and promotion of mental health and well-being, and the discrete conditions referred to within the Declaration text [1].

Officially adopted by the UN General Assembly on 15 December 2025 and noted at the 158th session of the WHO Executive Board on 3 February 2026, the final Declaration suggests that Member States have an appetite to prioritise actions for a ‘laundry list’ of NCDs. While the inclusivity of multiple NCDs brings attention to their burden, this approach is potentially problematic. First, it weakens an integrated NCD agenda where common risk factors and interventions that are effective across NCDs are prioritised, such as WHO’s ‘Best Buys’ [36], which is critical for the feasibility and sustainability of actions in LMICs. Second, conditions that are not explicitly listed or are ‘hidden in plain sight*’*, such as chronic pain and musculoskeletal conditions, are less likely to be considered by countries despite their unequivocal burden [5]. This context will likely destabilise implementation efforts for the WHO global technical products [29–32]. Third, the scope of the commitments may now become overwhelming for countries to implement, especially among those LMICs where resources are constrained, and progress on SDG 3.4 targets is already lagging, and where an integrated approach is most needed [4].

Should Member States demand list of priority NCDs beyond those listed in performance targets for SDG 3.4, then there is a strong argument that chronic pain and musculoskeletal conditions should be included as priority health conditions within NCD policy, programmes and monitoring frameworks. However, a more sustainable approach to reduce health loss across NCDs, particularly in LMICs, could be achieved through the integration of efforts to prevent and control chronic pain and mobility impairment within whole-of-government and whole-of-society responses to NCDs. Such a horizontal integration approach, rather than vertical NCD programmes, is more likely to be feasible in resource-constrained settings and would better enable integrated care for common co- and multi-morbid NCD presentations, where chronic pain and mobility impairment due to musculoskeletal conditions are common [16]. For example, this integrated approach is emphasised in the UN Decade of Healthy Ageing Plan of Action [15]. The Plan is framed around four action areas, where three are relevant to prevention and control of chronic pain and musculoskeletal conditions in older people (combatting ageism, supporting age-friendly environments, and integrated care) [37]. Considering practical service delivery and workforce implications for countries, this integrative approach is also supported by WHO technical products. These include the WHO Integrated Care for Older People model, which explicitly integrates musculoskeletal and pain care alongside loss of capacity in other body systems, rather than selective disease care [32], and the WHO Packages of Interventions for Rehabilitation (basic and condition-specific packages [30,38]).

Despite this missed opportunity for reducing the burden of NCDs through integrating prevention and control of chronic pain and mobility impairment related to musculoskeletal conditions with other NCDs, ongoing advocacy remains essential. Specifically, in planning global health targets beyond 2030, setting targets and co-creating actions beyond mortality reduction to address the expanding morbidity burden, will be essential to improving global population health, healthy life years and healthy ageing, while at the same time supporting countries to contain the escalating healthcare and indirect costs attributed to chronic pain and musculoskeletal health conditions [39–41]. Relevant indicators that embrace morbidity reduction should be co-created with countries and might encompass population health surveillance capability, gain of healthy life years (*i.e.* healthy life expectancy), service accessibility without financial hardship (*e.g.* access to medications, surgery, rehabilitation services) and risk factor mitigation (*e.g.* obesity, injury).
